# Study of Complexes of Tannic Acid with Fe(III) and Fe(II)

**DOI:** 10.1155/2019/3894571

**Published:** 2019-02-03

**Authors:** Zhaofeng Fu, Rui Chen

**Affiliations:** College of Chemistry and Chemical Engineering, Yunnan Normal University, Kunming 650500, China

## Abstract

UV-Vis absorption spectra of tannic acid were gained at pH 1.0∼9.0. Due to the pH value dependence of complex, the stoichiometry of tannic acid with iron ions was tested in buffer solution by the mole ratio method. The result suggests that the complex ratio of tannic acid to Fe(III) is 1 : 1 and to Fe(II) 3 : 1 in the carbonate buffer solution, and the complex ratio of iron-tannic complexes is 1 : 1 at pH 2.2. Due to the different color changes of tannic acid with iron ions in the coordination reactions, a tannic acid test paper was designed. The concentrations of Fe(III) more than 5.000 × 10^−6^ mol/L and the concentrations of Fe(II) more than 1.000 × 10^−5^ mol/L in aqueous solution can be detected by this test paper.

## 1. Introduction

In recent years, tannic acid, a natural plant product, has been widely applied in medicine [1–3], food [4], tanning [5], cosmetic [6], metallurgical, and other industries [7, 8]. Tannic acid, a kind of polyphenols, possesses the numerous phenolic hydroxyl groups in the structure, which make it possess excellent physical and chemical properties [9, 10] and remarkable biological and pharmacological activities [11–16]. Tannic acid can interact with metals [17, 18], proteins [19, 20], alkaloids [21], and polysaccharides [22] and perform several physiological and ecological effects [18, 23].

Iron ions play an important role in life processes [16]. In chemistry, several methods are used to identify Fe(III) and Fe(II) including the thiocyanate method [24, 25] and the sulfosalicylic acid method [26, 27]. Additionally, the pH test paper is often employed as a convenient way in assessing acidity in the experiment. A porphyrin-based test paper was applied to detect Fe(III) and Al(III) in aqueous solution [28] because of the coordination reactions and the resulting color changes between porphyrin and metal ions. Metal complexes of tannic acids usually show characteristically absorption bands in the visible region [17] that can be used as sensitive chromogenic sensors for detection of metal ions. Hence, the stoichiometry of tannic acid with an iron ion was detected in the carbonate buffer solution by the mole ratio method. The tactics to easily identify Fe(III) and Fe(II) in aqueous solution were provided by the tannic acid test paper.

## 2. Experimental

The experimental was carried out as described by Sungur and Uzar [16] and Li et al. [28] with little modifications. Solutions of tannic acid, FeSO_4_, and Fe_2_(SO_4_)_3_ were prepared with concentrations of 1.000 × 10^−4^ mol/L in aqueous solution, respectively. Solution of NH_2_OH·HCl was prepared with a concentration of 1.440 mol/L in aqueous solution. Buffer solution at pH 1.0 was prepared by HCl. Buffer solution at pH 2.2 was prepared by adding water in 4.20 g citric acid, 1.68 g sodium citrate, and 3.20 mL HCl and then dissolving and diluting them to 200 mL. Buffer solutions at pH 3.0∼6.0 were prepared by citric acid and sodium citrate, which were dissolved in 200 mL water.  Table 1 shows the amount of citric acid and sodium citrate at different pH values. Buffer solution at pH 8.0 was prepared by adding water in 0.12 g citric acid and 5.52 g Na_2_HPO_4_ and then dissolving and diluting them to 200 mL. Carbonate buffer solution at pH 9.0 was prepared by adding water in 16.75 g NaHCO_3_ and 5.40 g Na_2_CO_3_·10H_2_O and then dissolving and diluting them to 200 mL. Deionized water was used to prepare all solutions.

A strip of the filter paper (1.0 cm × 7.0 cm) was immersed in 1.000 × 10^−2^ mol/L tannic acid aqueous solution for 1 min and then dried in air. The tannic acid test paper was then obtained.

UV-Vis absorption spectra of sample solutions were acquired using a TU-1900 double beam ultraviolet-visible spectrophotometer (Purkinje General Instrument, China). The spectra were taken over the wavelength range of 200∼800 nm at room temperature in a 1 cm quartz cuvette. The correlation coefficient (*R*_2_) value for the UV calibration line is above 99.9%.

## 3. Results and Discussion

### 3.1. Stoichiometry of Tannic Acid-Iron Complexes

**Scheme 1:**
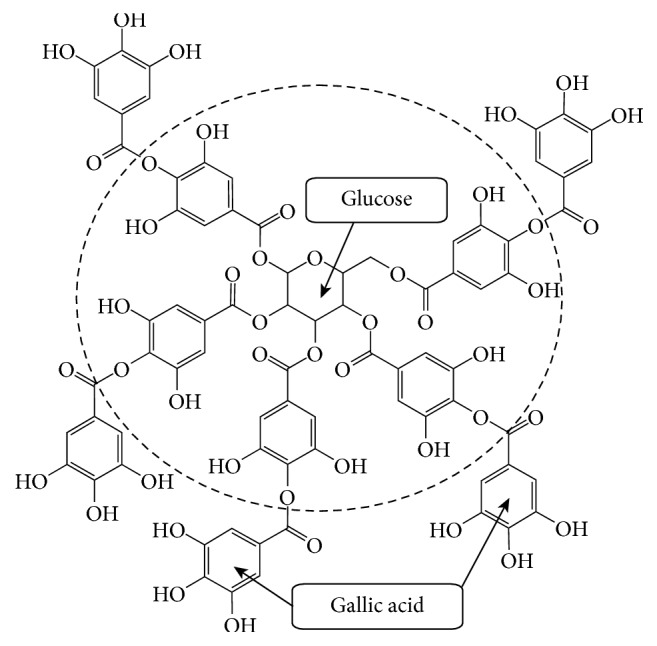
Chemical structure of tannic acid, a decagalloyl residue consisting of a center glucose molecule esterified at all five hydroxyl moieties with two gallic acids. The circle indicates pentagalloylglucose and the core structure of tannic acid.

**Figure 1:**
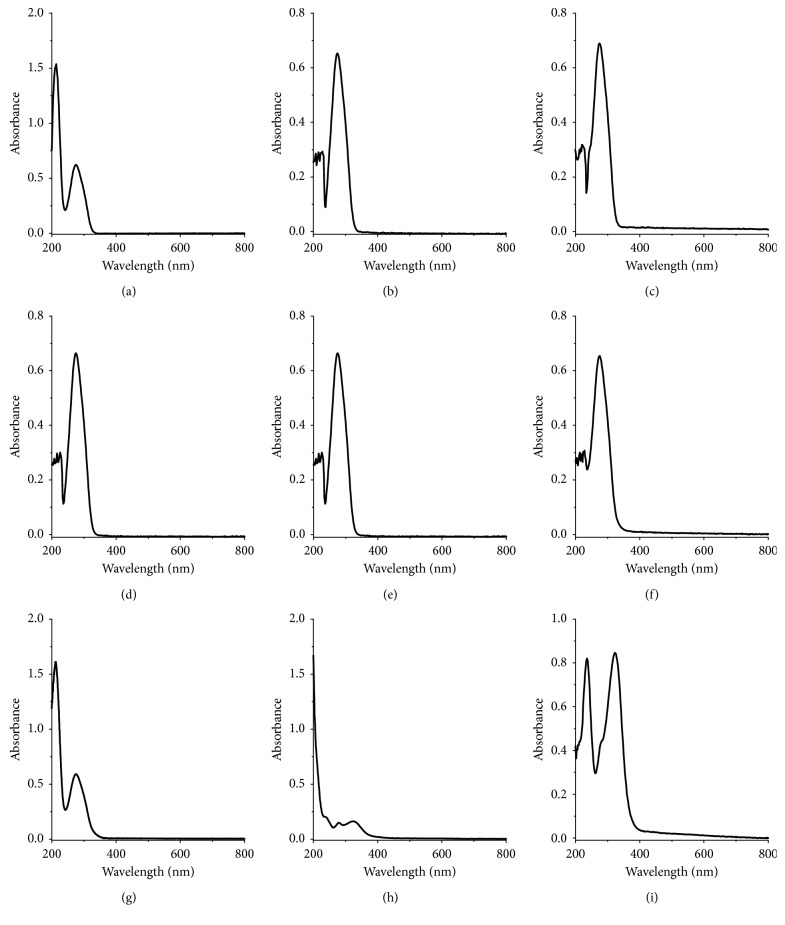
UV-Vis absorption spectra of 1.000 × 10^−5^ mol/L tannic acid at different pH values. (a) pH = 1.0. (b) pH = 2.0. (c) pH = 3.0. (d) pH = 4.0. (e) pH = 5.0. (f) pH = 6.0. (g) pH = 7.0. (h) pH = 8.0. (i) pH = 9.0.

Tannic acid is composed of a central glucose molecule derivatized at its hydroxyl groups with 10 galloyl residues (Scheme 1), which act as a strong UV absorbing chromophore and show intense absorption bands at 274 nm (Figure 1).

The protonated phenolic group is not a good ligand for the metal cation. However, once the phenolic group is deprotonated, an oxygen center will be generated [29]. The *peri* positions of phenolic hydroxyl groups present as oxygen anions and can react with metal ions to form stable five-numbered ring complexes. Although the third phenolic hydroxyl in gallic acids is absent from the coordination reaction, it can promote the delocalization of the lone pairs associated with the other two hydroxyl groups and stabilize the complex [30].

We discuss the effect of the pH value on the UV-Vis absorption spectra of tannic acid. 1 mL 1.000 × 10^−4^ mol/L tannic acid was dissolved and diluted to 10 mL in buffer solution for UV-Vis analysis. Figure 1 shows the UV-Vis absorption spectra of 1.000 × 10^−5^ mol/L tannic acid in buffer solution at different pH values. There are two intense absorption bands at 213 nm and 276 nm in the UV-Vis absorption spectra of tannic acid at pH 1.0. The absorption band at 213 nm disappears, and there is an intense absorption band at 276 nm in the UV-Vis absorption spectra of tannic acid at pH 2.0 to 6.0. The two bands at 213 nm and 276 nm appear in the UV-Vis absorption spectra of tannic acid at pH above 7.0. With the increase of pH, the absorption band at 276 nm becomes strong and that at 213 nm becomes weak. The result shows that the UV-Vis absorption spectra of tannic acid are strongly pH dependent.

**Figure 2:**
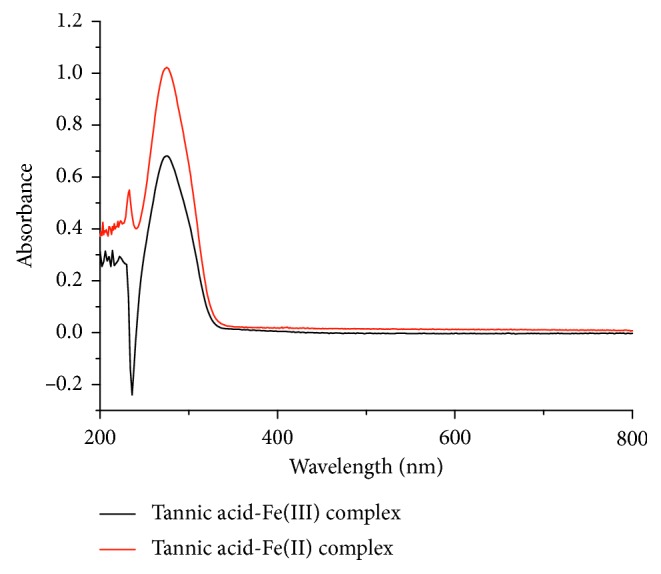
UV-Vis absorption spectra of the tannic acid-Fe(III) complex and tannic acid-Fe(II) complex at pH 2.2, respectively.

Due to the pKa value of tannic acid and the pH value dependence of complex [16, 29], the solution was chosen to adjust and control the pH value of the coordination reactions between tannic acid and iron ion. The UV-Vis absorption spectra of the tannic acid-Fe(III) complex and tannic acid-Fe(II) complex at pH 2.2 are remarkably different from that of tannic acid at the same pH value in Figure 2. The result shows that tannic acid can form complex with the Fe(III) and Fe(II) complex at pH 2.2.

**Figure 3:**
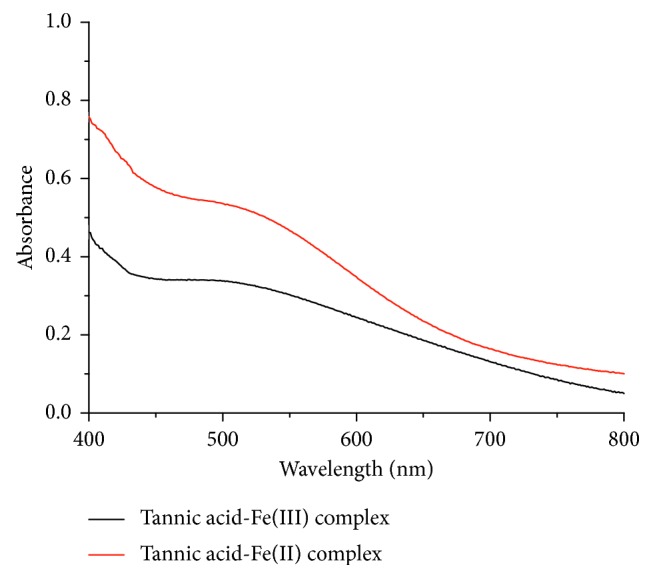
UV-Vis absorption spectra of tannic acid-Fe(III) complex and tannic acid-Fe(II) complex at pH 9.0, respectively.

It is observed that the reaction between tannic acid and Fe(III) in carbonate buffer solution forms yellow green complex and the reaction between tannic acid and Fe(II) in carbonate buffer solution forms magenta complex. In Figure 3, the absorption spectra of both complexes appear in the visible region including fine peaks over the wavelength range of 400–450 nm and wide peaks centered at 500 nm. Therefore, the absorbance of complexes at 500 nm was selected to determine the complex ratios of the complexes to avoid the interference of tannic acid by the mole ratio method.

Generally, the stoichiometry of the metal-ligand complexes can be determined by the mole ratio method, the slope-ratio method, the method of continuous variations, and the mobile equilibrium method. The mole ratio method is a procedure for determining the stoichiometry between two reactants by preparing solutions containing different mole ratios of two reactants [31]. A series of solutions were prepared in which the concentration of metal ions was held constant and 10 mL carbonate buffer solution was added to sample liquid. In addition, 1.0 mL NH_2_OH·HCl aqueous solution was added to the tannic acid and Fe(II) solution to avoid the oxidation of Fe(II) in air.

**Figure 4:**
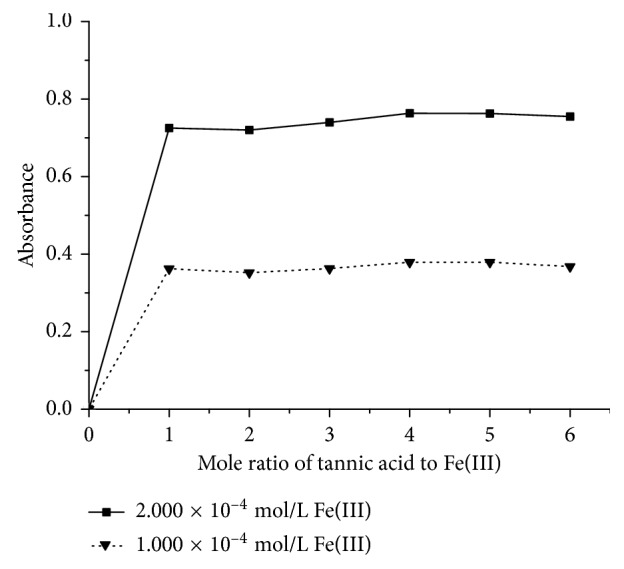
The mole ratio curves of the tannic acid-Fe(III) complex by the mole ratio method. The solid line denotes 2.000 × 10^−4^ mol/L Fe(III) in sample liquid. The dotted line denotes 1.000 × 10^−4^ mol/L Fe(III) in sample liquid.

**Figure 5:**
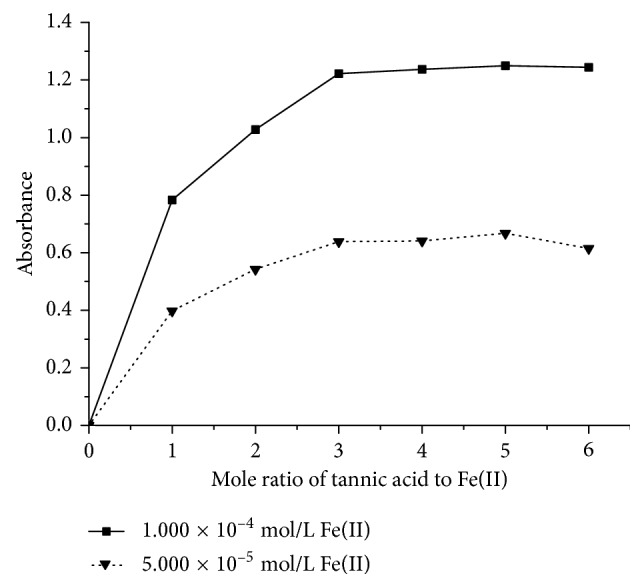
The mole ratio curves of the tannic acid-Fe(II) complex by the mole ratio method. The solid line denotes 1.000 × 10^−4^ mol/L Fe(II) in sample liquid. The dotted line denotes 5.000 × 10^−5^ mol/L Fe(II) in sample liquid.

In Figure 4, with the increase of the mole ratio of tannic acid to Fe(III), absorbance increases firstly and then tends to be constant. When the mole ratio of tannic acid to Fe(III) is more than 1 : 1, the curve becomes stable. The stoichiometry of the tannic acid-Fe(III) complex is 1 : 1; that is, the formula for the tannic acid-Fe(III) complex can be written as [(tannic acid)Fe]^3+^ at pH = 9.0. Similarly with the increase of the mole ratio of the tannic acid-Fe(II) complex, absorbance increases and then tends to be constant in Figure 5. The formula for the tannic acid-Fe(II) complex is [(tannic acid)_3_Fe]^2+^.

**Figure 6:**
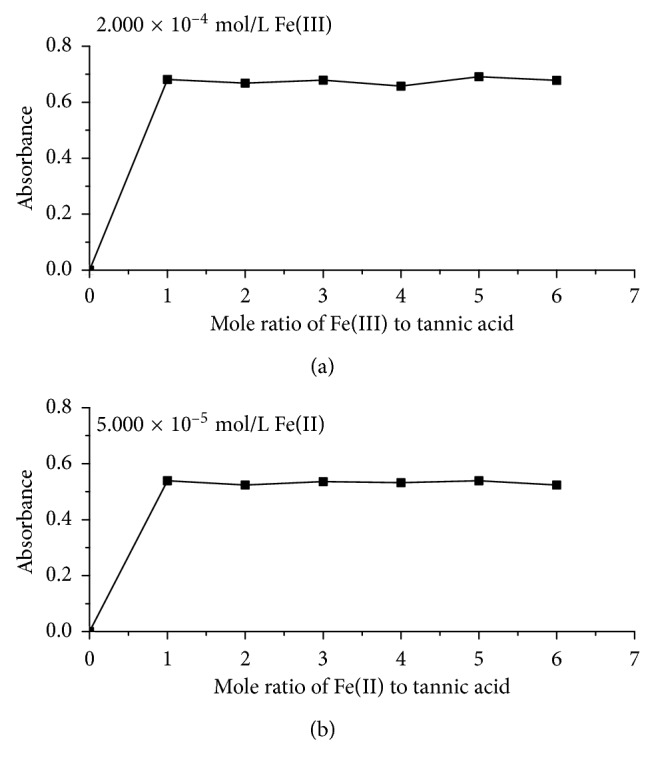
The mole ratio curves of tannic acid-Fe(III) (a) and tannic acid-Fe(II) (b) complexes by the mole ratio method.

In Figure 6, using the same method, we analyzed the mole ratios of tannic acid-Fe(II) and tannic acid-Fe(III) complexes, and the absorbance of which at 232 nm was selected to determine the complex ratios of the complexes to avoid the interference of tannic acid by the mole ratio method at pH 2.2. The result shows that the mole ratios of the tannic acid-Fe(III) complex and tannic acid-Fe(II) complex are 1 : 1 and 1 : 1; that is, the formula for them can be written as [(tannic acid)Fe]^3+^ and [(tannic acid)Fe]^2+^ at pH 2.2. The stoichiometry of iron-tannic complexes at pH 2.2 is in agreement with the previous finding [16].

### 3.2. Detection Limit of the Tannic Acid Test Paper

The coordination reaction between tannic acid with Fe(II) forms purple products in aqueous solution and with Fe(III) dark blue complex. Hence, we can indicate the existence of Fe(III) or Fe(II) according to the color changes of coordination reactions. Due to the facile identification feature of the test paper, we design the tannic acid test paper to differentiate iron ions.

Herein, we selected the 1.000 × 10^−2^ mol/L Fe(III) solutions as standard solution to determine the optimal concentration of tannic acid, which was used to make the tannic acid test paper. The optimal preparation condition of the tannic acid test paper is considered to immerse the filter paper in 1.000 × 10^−2^ mol/L tannic acid solution for 10 s before drying in air. By adding a drop of Fe^3+^ solution with the concentration of 1.000 × 10^−2^ mol/L, 1.000 × 10^−3^ mol/L, 1.000 × 10^−4^ mol/L, 1.000 × 10^−5^ mol/L, 5.000 × 10^−6^ mol/L, and 1.000 × 10^−6^ mol/L to the tannic acid test paper, the color of the test paper changed into dark blue, deep blue, clear blue, purple, lavender, and colorless, respectively (Figure 1S in Supporting Information). When a drop of Fe^2+^ solution with the concentration of 1.000 × 10^−2^ mol/L, 1.000 × 10^−3^ mol/L, 1.000 × 10^−4^ mol/L and 1.000 × 10^−5^ mol/L to the tannic acid test paper is added, the color of the test paper changed into modena, bluish violet, deep violet, and colorless, respectively (Figure 2S in Supporting Information). The result suggests that the test paper can identify the concentration of Fe(III) solution more than 5.000 × 10^−6^ mol/L and the concentration of Fe(II) solution more than 1.000 × 10^−5^ mol/L, which can be observed by naked eye.

## 4. Conclusions

The mole ratio method was applied to determine the formula for the complexes between tannic acid and iron ions in carbonate buffer solution at 500 nm by UV-Vis absorption spectra. The result shows that the stoichiometry between tannic acid and Fe(III) is 1 : 1 in the complex, tannic acid and Fe(II) is 3 : 1 at pH 9.0, and iron-tannic complexes is 1 : 1 at pH 2.2. The tannic acid test paper designed can detect the concentrations of more than 5.000 × 10^−6^ mol/L Fe(III) and more than 1.000 × 10^−5^ mol/L Fe(II) in aqueous solution. A facile and convenient method was offered to identify the iron ion by the tannic acid test paper. The strategy can be applied to identify other metal ions through color changes of the coordination reaction in several fields including medicine, food industry, environment, biology, metallurgy, and other industries. The studies of iron-mediated self-assembly and the structure of iron-tannic complexes are currently in progress in our laboratories.

## Figures and Tables

**Table 1:** The amount of citric acid and sodium citrate at different pH values.

pH value	Amount of citric acid (g)	Amount of sodium citrate (g)
3.0	3.91	0.41
4.0	2.75	2.3
5.0	1.72	3.47
6.0	0.80	4.76

## Data Availability

The data used to support the findings of this study are available from the corresponding author upon request.

## Supplementary Materials

Supplementary Materials Figure 1S: using the tannic acid test paper to identify 1.000 × 10^−2^ mol/L Fe^3+^ solution (a), 1.000 × 10^−3^ mol/L Fe^3+^ solution (b), 1.000 × 10^−4^ mol/L Fe^3+^ solution (c), 1.000 × 10^−5^ mol/L Fe^3+^ solution (d), 5.000 × 10^−6^ mol/L Fe^3+^ solution (e), and 1.000 × 10^−6^ mol/L Fe^3+^ solution (f), respectively. Figure 2S: using the tannic acid test paper to identify 1.000 × 10^−2^ mol/L Fe^2+^ solution (a), 1.000 × 10^−3^ mol/L Fe^2+^ solution (b), 1.000 × 10^−4^ mol/L Fe^2+^ solution (c), and 1.000 × 10^−5^ mol/L Fe^2+^ solution (d), respectively.
