# Supplementary material for: Study of Complexes of Tannic Acid with Fe(III) and Fe(II)
Source: J Anal Methods Chem. 2019 Feb 3;2019:3894571. doi: 10.1155/2019/3894571 (PMC6378047; doi:10.1155/2019/3894571)
Supplement: Supplementary Materials — Figure 1S: using the tannic acid test paper to identify 1.000 × 10^−2^ mol/L Fe3+ solution (a), 1.000 × 10^−3^ mol/L Fe3+ solution (b), 1.000 × 10^−4^ mol/L Fe3+ solution (c), 1.000 × 10^−5^ mol/L Fe3+ solution (d), 5.000 × 10^−6^ mol/L Fe3+ solution (e), and 1.000 × 10^−6^ mol/L Fe3+ solution (f), respectively. Figure 2S: using the tannic acid test paper to identify 1.000 × 10^−2^ mol/L Fe2+ solution (a), 1.000 × 10^−3^ mol/L Fe2+ solution (b), 1.000 × 10^−4^ mol/L Fe2+ solution (c), and 1.000 × 10^−5^ mol/L Fe2+ solution (d), respectively. [file 3894571.f1.doc]

**Supporting Information**

Study of Complexes Tannic Acid with Fe(Ⅲ) and Fe(Ⅱ)

**Zhaofeng Fu, Rui Chen***

*College of Chemistry and Chemical Engineering, Yunnan Normal University, Kunming 650500, China*

E-mail: [rui_chen888@hotmail.com](mailto:rui_chen888@hotmail.com)


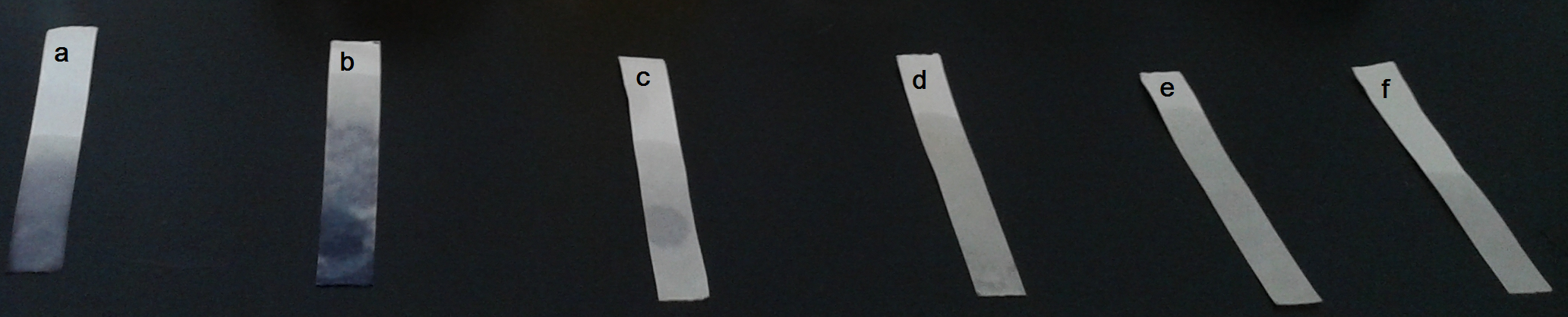


Figure 1S Using tannic acid test paper to identify 1.000×10^-2^ M Fe^3+^ solution (a), 1.000×10^-3^ M Fe^3+^ solution (b), 1.000×10^-4^ M Fe^3+^ solution (c), 1.000×10^-5^ M Fe^3+^ solution (d), 5.000×10^-6^ M Fe^3+^ solution (e), and 1.000×10^-6^ M Fe^3+^ solution (f), respectively.


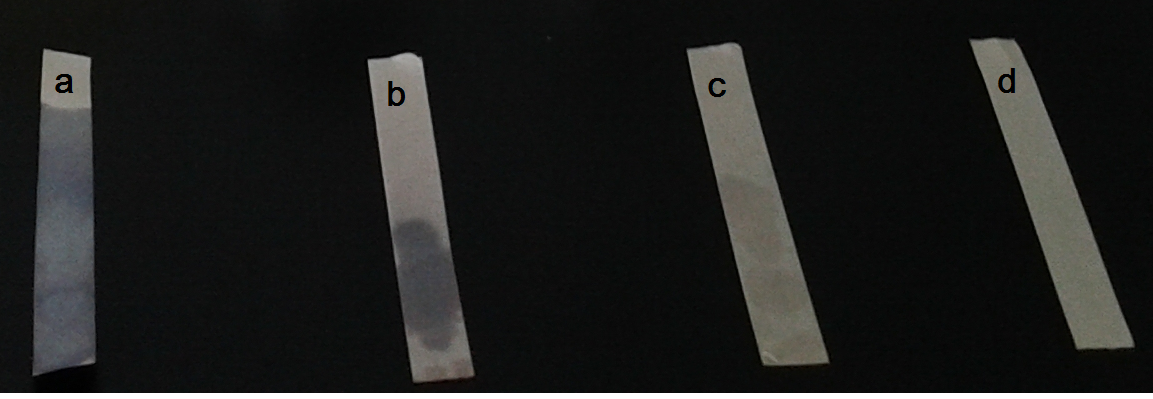


Figure 2S Using tannic acid test paper to identify 1.000×10^-2^ M Fe^2+^ solution (a), 1.000×10^-3^ M Fe^2+^ solution (b), 1.000×10^-4^ M Fe^2+^ solution (c), and 1.000×10^-5^ M Fe^2+^ solution (d), respectively.
